# Impact of newly diagnosed abnormal glucose regulation on long-term prognosis in low risk patients with ST-elevation myocardial infarction: A follow-up study

**DOI:** 10.1186/1472-6823-11-14

**Published:** 2011-07-29

**Authors:** Eva C Knudsen, Ingebjørg Seljeflot, Michael Abdelnoor, Jan Eritsland, Arild Mangschau, Carl Müller, Harald Arnesen, Geir Ø Andersen

**Affiliations:** 1 Center for Clinical Heart Research, Oslo University Hospital, Ullevål, Oslo, Norway; 2 Department of Cardiology, Oslo University Hospital, Ullevål, Oslo, Norway; 3 Center of Clinical Research, Unit of Epidemiology and Biostatistics, Oslo University Hospital, Ullevål, Oslo, Norway; 4 Center for Heart Failure Research, Oslo University Hospital, Ullevål, Oslo, Norway; 5 Faculty of Medicine, University of Oslo, Oslo, Norway; 6 Nuclear Medicine, Oslo University Hospital, Ullevål, Oslo, Norway

## Abstract

**Background:**

Patients with acute myocardial infarction and newly detected abnormal glucose regulation have been shown to have a less favourable prognosis compared to patients with normal glucose regulation. The importance and timing of oral glucose tolerance testing (OGTT) in patients with acute myocardial infarction without known diabetes is uncertain. The aim of the present study was to evaluate the impact of abnormal glucose regulation classified by an OGTT in-hospital and at three-month follow-up on clinical outcome in patients with acute ST elevation myocardial infarction (STEMI) without known diabetes.

**Methods:**

Patients (n = 224, age 58 years) with a primary percutanous coronary intervention (PCI) treated STEMI were followed for clinical events (all-cause mortality, non-fatal myocardial re-infarction, recurrent ischemia causing hospital admission, and stroke). The patients were classified by a standardised 75 g OGTT at two time points, first, at a median time of 16.5 hours after hospital admission, then at three-month follow-up. Based on the OGTT results, the patients were categorised according to the WHO criteria and the term abnormal glucose regulation was defined as the sum of impaired fasting glucose, impaired glucose tolerance and type 2-diabetes.

**Results:**

The number of patients diagnosed with abnormal glucose regulation in-hospital and at three-month was 105 (47%) and 50 (25%), respectively. During the follow up time of (median) 33 (27, 39) months, 58 (25.9%) patients experienced a new clinical event. There were six deaths, 15 non-fatal re-infarction, 33 recurrent ischemia, and four strokes. Kaplan-Meier analysis of survival free of composite end-points showed similar results in patients with abnormal and normal glucose regulation, both when classified in-hospital (p = 0.4) and re-classified three months later (p = 0.3).

**Conclusions:**

Patients with a primary PCI treated STEMI, without previously known diabetes, appear to have an excellent long-term prognosis, independent of the glucometabolic state classified by an OGTT in-hospital or at three-month follow-up.

**Trial registration:**

The trial is registered at http://www.clinicaltrials.gov, NCT00926133.

## Background

Patients hospitalised with acute myocardial infarction (MI) have a reported incidence of known type 2 diabetes between 10 and 20% [1]. Patients with type 2 diabetes and acute MI have a higher incidence of new cardiovascular events and higher in-hospital and long-term mortality rate compared to non-diabetic patients [2-4]. In diabetic patients with ST elevation myocardial infarction (STEMI), primary percutaneous coronary intervention (PCI) with adjunctive glycoprotein IIb/IIIa inhibition has been shown to be associated with improved prognosis compared to fibrinolytic treatment [5].

Several studies have reported that a substantial proportion of patients with acute MI, without known type 2 diabetes, have disturbed glucometabolic regulation [6,7]. In addition, an increased risk of cardiovascular morbidity and mortality has been demonstrated in patients with acute MI and newly detected abnormal glucose regulation [8], emphasizing the importance of diagnosing these patients at an early stage. Accordingly, the European guidelines on diabetes, pre-diabetes and cardiovascular diseases from 2007 recommend that these patients should be investigated with an oral glucose tolerance test (OGTT) [9]. The timing and importance of broad OGTT screening after an acute MI are, however uncertain, especially in patients subjected to modern treatment with primary PCI resulting in smaller infarct size and improved prognosis.

We have previously reported that patients with a primary PCI treated STEMI without known diabetes, have a high prevalence of undetected abnormal glucose regulation classified by an OGTT either in-hospital or three months later [10]. However, when repeating an OGTT in a stable condition at three-month follow-up the observed reproducibility of the early test was poor, only 54% of the patients remained in the same glucometabolic category after a repeated OGTT. The OGTT performed early after a STEMI did not provide reliable information on long-term glucometabolic state [10].

An additional rationale for an early OGTT in patients without known diabetes with a recent acute MI is to identify high-risk patients with poor prognosis. The aim of the present study was to investigate clinical outcome in patients with a primary PCI treated STEMI after exclusion of patients with known diabetes. Secondly, to evaluate whether abnormal glucose regulation classified by OGTT acutely in-hospital or in a stable condition at three-month follow-up was associated with poorer long-term prognosis after acute STEMI.

## Methods

### Study population

Patients with an acute STEMI without known diabetes admitted to the coronary care unit, Oslo University Hospital, Ullevål, Oslo, Norway were prospectively enrolled from November 2005 to May 2007. The cohort is described in detail elsewhere [10]. In brief, patients with a primary PCI treated STEMI were included if they were hemodynamically stable, without chest pain or nausea, age < 85 years and with serum creatinine < 200 umol/L. Patients with previously known type 2 diabetes, persistent hyperglycaemia or clinical signs of heart failure were excluded. Heart failure complicating acute MI was defined according to the ESC Guidelines from 2008 [11]. Patients with persistent hyperglycaemia were defined as patients with both admission plasma glucose >11 mmol/L and a fasting capillary glucose level > 8 mmol/L. A standardized 75 g OGTT (plasma glucose measurements at 0 and 120 min) was performed after an overnight fast [12]. Altogether 224 patients were included. The inclusion date was defined as the date when the primary PCI was performed.

Based on the result of the OGTT, the patients were glucometabolically classified according to the WHO guidelines from 2005 into one of the following categories [13] (glucose levels given in mmol/L):

Normal Glucose Tolerance (NGT) = OGTT (0 min) < 6.1 and OGTT (2 h) < 7.8

Impaired Fasting Glucose (IFG) = OGTT (0 min) ≥ 6.1 **<**7.0 and OGTT (2 h) **<**7.8

Impaired Glucose Tolerance (IGT) = OGTT (0 min) **<**7.0 and OGTT (2 h) ≥7.8 < 11.1

Type 2 diabetes (T2DM) = OGTT (0 min) ≥7.0 and/or OGTT (2 h) ≥11.1.

The term abnormal glucose regulation was defined as the sum of IFG, IGT and T2DM.

This was an observational study. The results of analyses of blood glucose, either fasting or during OGTT were available for the treating physician at all time. Hyperglycaemia, if present, was treated at the physician's decision according to local, hospital practice. Patients were returned back to the referring hospital with a hospital record, which included information about the OGTT results. The responsible physician was asked to perform plasma glucose analyses repeatedly and start treatment if indicated. A diagnosis of diabetes was not based on the early OGTT performed during acute illness. The results of the OGTT and fasting glucose value were communicated to the general practitioners (GP) responsible for the patient. If an asymptomatic patient were diagnosed with type 2 diabetes at both occasions (in-hospital and at follow-up) the GP was contacted by phone and asked to confirm the diagnosis and initiate lifestyle changes and medical treatment according to national guidelines.

STEMI was defined according to the universal definition of myocardial infarction as typical rise and fall of the cardiac biomarker troponin T with at least one value above the 99th percentile of the upper reference limit in patients presenting with symptoms of ischemia together with new ST elevation at the J-point in two contiguous leads with the cut-off points: 0.2 mV in men or 0.15 mV in women in leads V2-V3 and/or 0.1 mV in other leads or new left bundle-branch block [14]. The median time from onset of chest pain to balloon (PCI) was 219 minutes (140, 378).

The Regional ethics committee approved the study and all patients provided written and oral informed consent.

### Laboratory methods

After overnight fasting, blood samples for routine analysis including glucose and HbA1c were drawn and analysed by use of conventional methods. Serum cardiac specific Troponin T (TnT) was measured by electrochemiluminescence technology for quantitative measurement (3^rd ^generation TnT, Elecsys 2010, Roche, Mannheim, Germany). The lower detection limit of the assay is 0.01 ug/L with a recommended diagnostic threshold of 0.03 ug/L. The inter-assay coefficient of variation was 7%.

Insulin was measured by a competitive radioimmunoassay (RIA) kit (Linco Research, Inc, ST. Charles, MO, US) and C-peptide was determined by Immulite 2000 (Diagnostic Product Corporation, Los Angeles, CA, US). Proinsulin was measured by an enzyme immunoassay (kit) from DRG instruments (Gmbtt, Marburg. Germany). The homeostasis model assessment of insulin resistance (HOMA-IR) was calculated as fasting serum insulin (in pmol/L) multiplied with fasting plasma glucose (in mmol/L) and divided by 135 [15].

### Follow up

At three-month follow-up, clinical examination and a repeated OGTT were performed.

In addition, left-ventricular (LV) function and infarct size were assessed at rest at follow-up. Perfusion imaging was performed according to ECG-gated SPECT after injection of ^99^m-tetrofosmin (Myoview™, Amersham Health, UK). An Exeleris processing station (GE Medical Systems) with 4D-MSPECT™ software (University of Michigan), was used for processing of all recordings and assessment LV volumes, LV ejection fraction (LVEF) and infarct size (proportion perfusion defect) expressed as percent of LV mass [16].

In June 2009, at a median follow up time of 33 months, the patients were contacted and interviewed by telephone, regarding their health status, and any hospital admissions. Closing date was set to August 1st 2009. None of the patients were lost to long-term follow-up, but only 201 patients attended the three-month follow-up examination. In case of re-hospitalisation, hospital records were collected from the patients' community hospitals.

The primary endpoint of the study was defined as the composite of the first event of non-fatal myocardial re-infarction, recurrent ischemia causing hospital admission, stroke, and all cause mortality. An end-point committee classified the end-points according to the hospital records, and death certificates were obtained from the Norwegian Death Certificate Registry.

### Statistics

Continuous variables are presented as median and 25, 75 percentiles and categorical variables as proportions. Due to skewness in most of the measured variables, non-parametric statistics was used throughout. Differences among groups were analysed by Mann-Whitney test for continuous variables and the Chi square test for categorical data.

Univariate and multivariate analyses of this dynamic cohort with censored data (Kaplan-Meier survival, Log-Rank test and Cox proportional hazard models) were performed. Potential confounders that were associated with clinical outcome or abnormal glucose regulation with a p-value <0.2, were included in the model. The following risk factors were included in a stratification model; age, gender, treated hypertension at admission, previous myocardial infarction, smoking, body mass index, elevated triglycerides and cholesterol, TnTmax, and infarct size expressed as percent of ventricular mass. Adjustment for the potential confounders identified by the model was performed [17] and the follow-up time in patients years was included. The STROBE guidelines were followed [18].

A two-sided p value <0.05 was considered statistically significant. All analyses were performed using Epi-info software, 2005, version 3.3.2.

## Results

In-hospital OGTT was successfully performed in all patients (n = 224) and categorised as described. As previously reported [10], the prevalence of abnormal glucose regulation in-hospital was 47% (n = 105), of whom 24 patients (23%) (or 11% of the total population, n = 224) were classified with newly detected type 2 diabetes.

The OGTT was repeated in 201 patients three months after the acute STEMI. Twenty-three patients did not perform the repeated OGTT due to death (n = 1) or unwillingness (n = 22). The 22 patients who were unwilling to participate at three-month follow-up did, however, take part in the interview by telephone in June 2009. Five out of 22 patients reported a new clinical event during follow-up, one patient within the first three months, and four patients between three-month and (median time) 33 months follow-up. The number of patients with normal and abnormal glucose regulation classified in-hospital was 9 and 13, respectively, and two out of five with a new clinical event had abnormal glucose regulation. The prevalence of abnormal glucose regulation in the total population at three-month follow-up was reduced to 25% (n = 50), of whom 10 patients (20%) (or 5% of the total population, n = 201) were classified as having type 2 diabetes [10].

### Clinical and biochemical characteristics

Clinical characteristics of the study population according to the glucometabolic classification in-hospital and at three-month follow-up are shown in Table 1. Notably, patients with abnormal compared to normal glucose regulation were older, a higher proportion were women, and they had larger myocardial infarct size measured as % of left ventricular mass three months later. Significantly higher levels of HbA1c, insulin, proinsulin, and HOMA-IR (measured in-hospital and at three-month follow-up) were found in patients with newly detected abnormal glucose regulation independently of the classification time point (Table 2).

**Table 1:** Clinical characteristic of patients with STEMI according to glucometabolic classifications by an OGTT in-hospital and after three months

	In-hospital			At three-month follow-up		
	**NGR (n = 119)**	**AGR (n = 105)**	**P**	**NGR (n = 151)**	**AGR (n = 50)**	**P**

Age (years)	55 (50,63)	60 (54,71)	<0.001	57 (51, 65)	61 (52, 72)	0.04
Men	106 (89.1)	79 (75.2)	<0.01	129 (85.4)	36 (72.0)	0.03
Previous disorders						
Treated dyslipidaemia	11 (9.2)	9 (8.6)	0.86	15 (9.9)	4 (8.0)	0.47
Treated hypertension	25 (21)	33 (31.4)	0.32	38 (25.2)	17 (34.0)	0.23
Previous acute MI	9 (7.6)	7 (6.7)	0.08	10 (6.6)	4 (8.0)	0.48
Previous angina pectoris	5 (4.2)	2 (1.9)	0.33	6 (4.0)	1 (2.0)	0.45
Current smoker	65 (54.6)	44 (41.9)	0.06	67 (44.4)	24 (48.0)	0.66
Body mass index (kg/m^2^)	25.9 (24.5, 28.7)	26.1 (24.3, 28.6)	0.82	26.1 (24.4, 28.5)	25.7 (24.1, 28.7)	0.73
Waist circumference	100 (94, 107)	99 (94, 107)	0.86	101 (94, 107)	98 (94, 107)	0.92
Cholesterol (mmo/L)	5.2 (4.5, 5.8)	5.1 (4.4, 5.8)	0.52	4.1 (3.8, 4.6)	4.0 (3.6, 4.8)	0.43
LDL-cholesterol (mmol/L)	3.4 (2.7, 4.2)	3.3 (2.7, 4.0)	0.54	2.2 (1.8, 2.6)	2.1 (1.7, 2.6)	0.42
Triglycerides (mmo/L)	1.4 (1.0,1.8)	1.2 (0.9,1.8)	0.11	1.2 (0.9, 1.6)	1.2 (1.1, 1.6)	0.64
						
Stents	114 (95.8)	101 (96.2)	0.88	143 (94.7)	49 (98.0)	0.30
One-vessel disease	78 (65.5)	61 (58.1)	0.25	92 (60.9)	30 (60.0)	0.91
Left main disease	47 (39.5)	42 (40.0)	0.94	58 (38.4)	21 (42.0)	0.65
IIb/IIIa inhibitors	37 (31.1)	42 (40.0)	0.17	54 (35.8)	19 (38.0)	0.78
TnT peak value (ug/L)	4.47 (2.39, 7.32)	5.63 (2.78,10.89)	0.03	4.85 (2.45, 8.31)	4.97 (2.38, 10.25)	0.47
						
Measured at three-month.						
LVEF^1 ^(%)	64 (57, 70)	62 (52,69)	0.13	63.5 (56, 70)	63.5 (51, 69)	0.47
Infarct size^2 ^(%)	9 (0, 25)	20 (4.5, 32)	<0.001	13 (0, 27)	21.5 (0, 43)	0.04
						
Medication at discharge from CCU and after three months.						
Aspirin	119 (100)	105 (100)		150 (99.3)	50 (100)	0.57
Clopidogrel	118 (99.2)	104 (99.0)	0.93	143 (94.7)	46 (92.0)	0.49
β-blockers	96 (80.7)	85 (81.0)	0.96	142 (94.0)	46 (92.0)	0.61
Lipid lowering agents	118 (99.2)	103 (99.1)	0.49	146 (96.7)	48 (96.0)	0.82
ACEIs	19 (16)	17 (16.2)	0.96	52 (34.4)	17 (34.0)	0.96
ARBs	8 (6.7)	10 (9.5)	0.44	21 (13.9)	4 (8.0)	0.27

Data are presented as median values (25, 75 percentiles) or proportions.

ACEIs: angiotensin-converting enzyme inhibitors, AGR: abnormal glucose regulation, acute MI: acute myocardial infarction, ARBs: angiotensin receptor blockers, CCU: coronary care unit, NGR: normal glucose regulation, TnT peak level: serum peak level of cardiac specific Troponin T. ^1,2^Measured at three months by SPECT analysis, ^1^LVEF: left ventricular ejection fraction, ^2^infarct size in % of left ventricular mass.

**Table 2:** Biochemical variables in patients according to glucometabolic status classified by an OGTT in-hospital and at three-month

	NGR (in-hospital) (n = 119)	AGR (in-hospital) (n = 105)	P	NGR (at three-month) (n = 151)	AGR (at three-month) (n = 50)	P
HbA1c (%)	5.5 (5.3, 5.7)	5.6 (5.4, 5.8)	<0.01	5.5 (5.4, 5.7)	5.8 (5.5, 6.2)	<0.0001
Insulin (pmol/L)	66 (46, 87)	73 (53,108)	0.03	63 (39, 82)	75 (47, 112)	0.02
C-peptid (pmol/L)	788 (638, 1026)	1026 (728, 1241)	<0.001	834 (636, 1077)	893 (728, 1129)	0.11
Proinsulin (pmol/L)	6.8 (4.7, 10.4)	8.6 (5.8, 13.9)	0.02	5.4 (4.3, 8.5)	7.5 (5.4, 10.3)	0.01
HOMA-IR (mU mmol^-1 ^L^-1^)	2.45 (1.63, 3.36)	3.00 (2.11, 4.92)	<0.001	2.20 (1.45, 3.23)	3.15 (2.08, 4.99)	<0.01
Proinsulin: insulin ratio	0.12 (0.08, 0.19)	0.13 (0.08, 0.18)	0.87	0.10 (0.07, 0.15)	0.12 (0.07, 0.18)	0.41

Data are presented as median values (25, 75 percentiles)

AGR: abnormal glucose regulation, NGR: normal glucose regulation.

When comparing patients with and without new clinical events, there were no significant differences in clinical or biochemical characteristics (data not shown).

Glucose lowering medication was not introduced in-hospital or during the three-month follow-up period in any of the patients.

### Follow-up

The median follow-up time for clinical events was 33 months (27, 39). The long-term clinical outcome was excellent with low event-rate. Of the total population (n = 224), 58 (26%) patients experienced a new clinical event including 33 patients with recurrent ischemia. Six patients died, two of myocardial re-infarction, one of cancer, one of an infectious disease with multiorgan failure, and two died without known reason. Fifteen patients experienced a non-fatal re-infarction. Table 3 summarises all first events in relation to the glucometabolic status classified in-hospital.

**Table 3:** Number of first clinical events according to glucometabolic status classified by an OGTT in-hospital in patients (n = 224) with STEMI

Type of event	NGR (n = 119)	IFG (n = 12)	IGT (n = 69)	DM (n = 24)	Total
Death	1	1	4	0	6
Non-fatal re-infarction	9	0	5	1	15
Recurrent ischemia	20	0	8	5	33
Stroke	4	0	0	0	4
SUM	34	1	17	6	58

NGR: normal glucose regulation, IFG: impaired fasting glucose, IGT: impaired glucose tolerance, DM: type 2 diabetes mellitus.

**Figure 1:**
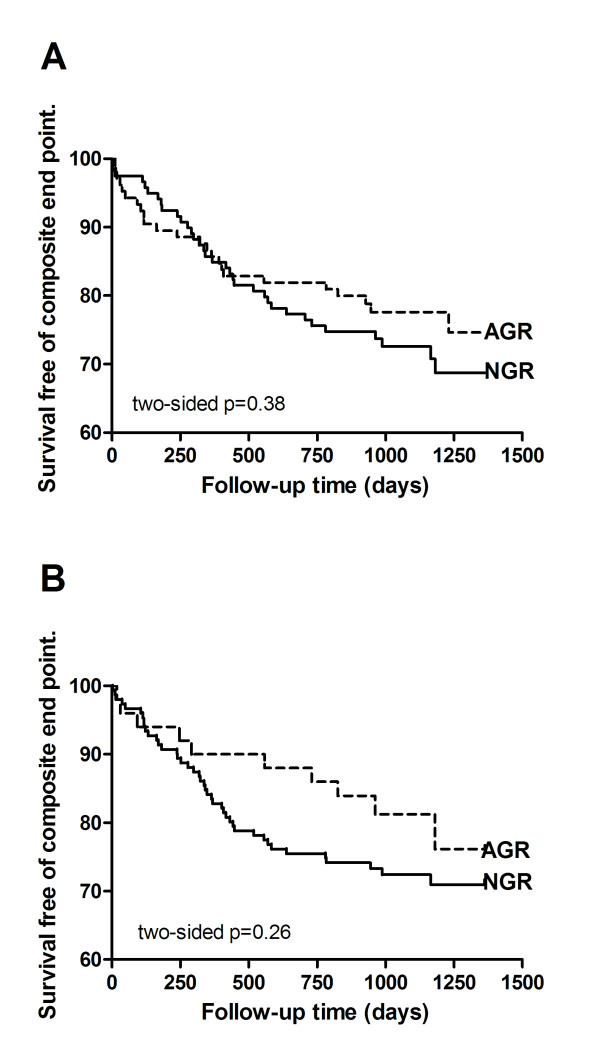
**Kaplan-Meier curves, survival free of composite end-point for patients with normal (NGR) and abnormal glucose regulation (AGR) classified by an OGTT in-hospital (A) and after three months (B)**.

The Kaplan-Meier curves for patients with normal (n = 119) or abnormal glucose regulation (n = 105) classified by an OGTT in-hospital are shown in Figure 1A. The probability of remaining free from a new clinical event did not differ between the two groups (Log-Rank, p = 0.4). In a multivariate analysis adjusting for potential confounders the association remained non-significant between the groups (adjusted HR 0.81 (95% CI 0.48-1.38), p = 0.44).

Long-term prognosis was also compared between groups of patients classified into normal (n = 151) and abnormal glucose regulation (n = 50) based on an OGTT performed in a stable condition at three-month follow-up. No significant difference in survival free of composite end-point was found between the groups (Figure 1B, Log-Rank, p = 0.3). Adjusting for potential confounders did not change the results significantly (adjusted HR 0.68 (95% CI 0.34-1.36), p = 0.27). Nine patients experienced a new clinical event before the OGTT classification was performed at three-month. Among these nine patients, seven and two patients were classified into the abnormal and normal glucose regulation group, respectively, based on the in-hospital OGTT.

## Discussion

Our main result was that stable STEMI patients without previously known diabetes have an excellent long-term prognosis independently of newly detected abnormal glucose regulation made by an OGTT screening either in-hospital or at three-month follow-up.

Large prospective studies have demonstrated both admission and fasting blood glucose levels to be independent predictors of long-term mortality in non-diabetic acute MI patients whether treated by primary PCI or not [19-24]. However, data evaluating the prognostic importance of an OGTT performed during or early after an acute MI in patients without previously known diabetes are scarce. The timing of testing patients for abnormal glucose regulation after an acute MI is also uncertain. Recommendation of early screening of acute MI patients with an OGTT in order to detect high-risk patients in the recent European guidelines is mainly based on the follow-up study of the GAMI trial [8,9]. In this follow-up study, abnormal glucose regulation classified by an OGTT day four or five post-MI was associated with worse clinical outcome during a median follow-up time of about three years [8].

Our study is the second study (after the GAMI study) to evaluate early OGTT during an acute STEMI as a prognostic tool, in patients without previously known diabetes, and the first study who compared the prognostic value of an OGTT based classification in-hospital and after three months. The low event rate in the present study prevents firm conclusions about the prognostic value of OGTT screening in patients with acute STEMI. There was a non-significant trend towards increased hazard in patients with abnormal vs. normal glucose regulation and a real difference between groups may have been detected if the sample size was larger. However, our results are not in line with the similar sized GAMI study and may challenge the present guidelines, which emphasize the importance of early testing of all patients with acute MI, especially in the modern era of acute revascularization of STEMI patients.

The excellent prognosis found in our study, regardless of the glucometabolic status, may partly be explained by the low proportion of patients classified with type 2 diabetes in-hospital and at three-month (11% and 5%, respectively). Nevertheless, patients classified with abnormal compared to normal glucose regulation in-hospital and at three-month follow-up had significantly higher fasting values of HbA1c, circulating levels of insulin and proinsulin, and higher HOMA-IR score when measured both acutely and in a stable condition. These results correspond to another trial with acute MI patients showing that HbA1c and proinsulin were in the lowest range in patients with normal glucose regulation, intermediate in patients with impaired glucose tolerance, and highest in patients with type 2 diabetes [25]. The present results suggest that the abnormal glucose regulation found in these STEMI patients not only seem to be a transient stress-induced response, but also may be a result of underlying insulin resistance.

The overall mortality rate in the present study was 2.7%. None died in-hospital and only one patient died during the first 30 days. Nine patients (7 with abnormal glucose regulation) experienced a new clinical event before the OGTT classification was performed at three-month follow-up. A low overall incidence of clinical events was found during the first three months of follow-up, suggesting that a early OGTT based classification of the glucometabolic status in order to identify high risk patients with worse prognosis is possibly of limited importance in low risk patients with a primary PCI treated STEMI.

The low overall incidence of clinical events reported in our study may partly be explained by the inclusion criteria. Unstable patients with cardiogenic shock, renal failure, ongoing chest pain, nausea and persistent hyperglycaemia were excluded from the study, probably making a selection bias towards more glucometabolically normal patients with better prognosis.

Furthermore, the patients included were relatively young and previous studies have shown a close relationship between newly diagnosed abnormal glucose regulation and age [8,26-28]. Additionally, a high proportion of our patients were diagnosed with single-vessel disease during coronary angiography [10], which may have contributed to the low event-rate observed in both groups.

Systematic use of recommended treatment (evidenced based medications and revascularisation) has been shown to have a favourable impact on one-year prognosis in patients with diabetes and coronary artery disease [29]. Patients in the present study were all treated by primary PCI in addition to a high proportion of patients on evidence based secondary prevention. All patients were treated according to guidelines regardless of glucometabolic status and this may explain the excellent prognosis observed in this STEMI population. Transient hyperglycaemia in patients with acute MI without known diabetes is common and is associated with worse outcome [19,24]. However, it has been difficult to prove that glucose control by insulin-glucose infusion [30] or insulin-glucose-potassium infusion [31], improve survival in patients with acute MI.

The European guidelines on diabetes, pre-diabetes, and cardiovascular diseases recommend that patients with cardiovascular disease without known diabetes should be investigated with an OGTT [9], but whether the OGTT should be performed early after a first cardiovascular event or later in a stable condition is not defined. The present results suggest that OGTT screening of patients with acute STEMI without known diabetes should be performed in a stable condition during the post-MI follow-up.

### Study limitations

The present study has certain limitations such as a possible selection bias towards more glucometabolically normal patients due to the exclusion of patients who were hemodynamically unstable, had severe renal failure and persistent hyperglycaemia. Accordingly, patients included, were somewhat younger than expected from a general STEMI population, had relatively few comorbidities and mainly one-vessel disease. It is possible that different results would be obtained in older MI patients since high age has been shown to be associated with ischemia related hyperglycaemia and poor glycemic control [28].

All aspects taken together may explain the overall low incidence of clinical events during the follow-up period, but may also reflect the advances in modern treatment of STEMI with primary PCI in all patients and optimal post-infarction treatment. The association between abnormal glucose regulation classified by an OGTT and clinical outcome should be further investigated in forthcoming studies including primary PCI treated STEMI patients, with a prolonged follow-up period, using major cardiovascular events as a primary end-point.

## Conclusions

Patients with a primary PCI treated STEMI, without previously known diabetes, appear to have an excellent long-term prognosis, independent of the glucometabolic state classified by an OGTT in-hospital or at three-month follow-up.

## Competing interests

The authors declare that they have no competing interests.

## Authors' contributions

ECK performed the statistical analysis and drafted the manuscript.

MA made substantial contribution with statistical analysis. GØA contributed with the conception and design of the study. CM analysed the SPECT data. ECK, IS, MA, JE, AM, HA and GØA participated in the study design and interpretation and revised the manuscript critically for important intellectual content. All authors read and approved the final manuscript.

## Pre-publication history

The pre-publication history for this paper can be accessed here:

http://www.biomedcentral.com/1472-6823/11/14/prepub
